# Petroselinum Crispum is Effective in Reducing Stress-Induced Gastric Oxidative Damage

**DOI:** 10.4274/balkanmedj.2015.1411

**Published:** 2017-01-05

**Authors:** Ayşin Akıncı, Mukaddes Eşrefoğlu, Elif Taşlıdere, Burhan Ateş

**Affiliations:** 1 Doğu Fertil IVF Center, Malatya, Turkey; 2 Department of Histology and Embryology, Bezmialem Vakıf University Faculty of Medicine, İstanbul, Turkey; 3 Department of Chemistry, İnönü University Faculty of Science and Art, Malatya, Turkey

**Keywords:** Lansoprazole, gastric oxidative damages, parsley

## Abstract

**Background:**

Oxidative stress has been shown to play a principal role in the pathogenesis of stress-induced gastric injury. Parsley *(Petroselinum crispum)* contains many antioxidants such as flavanoids, carotenoids and ascorbic acid.

**Aims:**

In this study, the histopathological and biochemical results of nutrition with a parsley-rich diet in terms of eliminating stress-induced oxidative gastric injury were evaluated.

**Study Design:**

Animal experimentation

**Methods:**

Forty male Wistar albino rats were divided into five groups: control, stress, stress + standard diet, stress + parsley-added diet and stress + lansoprazole (LPZ) groups. Subjects were exposed to 72 hours of fasting and later immobilized and exposed to the cold at +4 degrees for 8 hours to create a severe stress condition. Samples from the animals’ stomachs were arranged for microscopic and biochemical examinations.

**Results:**

Gastric mucosal injury was obvious in rats exposed to stress. The histopathologic damage score of the stress group (7.00±0.57) was higher than that of the control group (1.50±0.22) (p<0.05). Significant differences in histopathologic damage score were found between the stress and stress + parsley-added diet groups (p<0.05), the stress and stress + standard diet groups (p<0.05), and the stress and stress + LPZ groups (p<0.05). The mean tissue malondialdehyde levels of the stress + parsley-added group and the stress + LPZ group were lower than that of the stress group (p<0.05). Parsley supported the cellular antioxidant system by increasing the mean tissue glutathione level (53.31±9.50) and superoxide dismutase (15.18±1.05) and catalase (16.68±2.29) activities.

**Conclusion:**

Oral administration of parsley is effective in reducing stress-induced gastric injury by supporting the cellular antioxidant defence system.

Stress, one of the biggest problem of our era, is an important factor that underlies many diseases. Reactive oxygen species (ROS) have been identified as playing a prominent role in the pathogenesis of ulcerative damage induced by stress (1). Stress has been shown to accelerate the formation of ROS (2,3,4). Free oxygen radicals are highly reactive and attack all of the cellular components such as cell lipids, proteins, carbohydrates and DNA (5,6). Antioxidant nutrients are important for the body for protection against ROS. In fact, the cells of the body are capable of producing enzymatic antioxidants including superoxide dismutase (SOD), catalase (CAT) and glutathione peroxidase (GSH-Px) as well as non-enzymatic antioxidants including GSH. SOD scavenges superoxide (O_2_•−) whereas CAT and GSH-Px scavenge hydrogen peroxide (H_2_O_2_) (7,8,9).

H_2_ receptor blockers and proton pump inhibitors (PPIs) are drugs introduced in clinical use in the treatment of gastric ulcers. PPIs, e.g. omeprazole, lansoprazole (LPZ), pantoprazole and esomeprazole, are the most effective inhibitors of gastric acid secretion. LPZ is metabolized to sulfonamide derivatives in parietal cells. The resultant metabolites inactivate the H+/K+ ATPase enzyme system, and block the formation of acid by preventing the migration of the H+ ions into the lumen (10,11). LPZ can also scavenge free oxygen radicals and thereby maintain the gastric mucosal epithelium and vascular endothelium (12). In cases of peptic ulcers, a 30 mg/day dose for 8 weeks is recommended for healing (13).

Numerous studies have been performed in order to evaluate the efficacy of many nutrients of antioxidant properties against stress-induced tissue damage. Parsley *(Petroselinum crispum)* is an aromatic herb that has been used to give flavour and odour to dishes and salads for centuries (14,15,16,17). In addition, *Petroselinum crispum* is now planted throughout the world due to its usage in the food industry, perfume manufacturing, soaps and creams (18,19). Parsley is rich in several antioxidants including volatile oils (apiol, limonene and eugenol), flavonoids (luteolin, apigenin glycosides and quercetin), carotenoids, ascorbic acid, tocopherol, tannins, sterols, vitamins A, C and K, potassium, calcium and magnesium (14,15,16,17,20,21). Due to its flavonoid, carotenoid and ascorbic acid content, it is a potent free radical scavenger (22). Flavonoids have been shown to be effective in the treatment of gastric ulcers (23,24). Parsley was reported to scavenge the free radicals OH- and 2,2-diphenyl-1-picrylhydrazil, and thus to reduce lipid peroxidation (25).

A variety of experimental animal models have been developed to generate stress ulcers. The most commonly used methods are immobilization, cold exposure and swimming. In our study, we tried to generate a severe stress condition by applying starvation, immobilization and cold. We aimed to investigate stress-induced gastric mucosal alterations and the efficacy of parsley in comparison to LPZ via histological and biochemical methods.

## MATERIALS AND METHODS

### Experimental protocol

In this study, 40 adult male Wistar albino rats were used. Animals were fed with standard rat chow and tap water ad libitum and were maintained in a 12 h light/12 h dark cycle at 21º.

Subsequently the rats were randomly divided into five groups (each containing eight animals). The first group represented intact control. The rats from the second, third, fourth and fifth groups were exposed to starvation for 72 hours. At the end of the starvation period they were immobilized and kept at 4 ºC for 8 hours. The rats from the stress group (2nd group) were decapitated after the stress exposure. Animals from the stress + standard diet group (3rd group) were fed with standard rat chow for 7 days. Animals from the stress + parsley (Petrocelinum crispum; herbarium number: INU1199-2013 from XXXX University, Faculty of Art and Science, Department of Botany)-added diet group (4th group) were fed with a diet in which a 28 g/kg body weight/day dose of parsley (40%) was added to the standard diet for 7 days. Finally, rats from the stress + LPZ group (5th group) were administered a 0.5 mg/kg/day dose of LPZ (LPZ 30 mg, Nobel, Düzce, Turkey) dissolved in 4 mL of normal saline via gavages for 7 days.

The rats were sacrificed by cervical dislocation at the end of the experiment. The stomachs were removed and opened by cutting along the lesser curvature. A half portion of the body of the stomach was processed for histological evaluation, and the other half for biochemical analysis.

The experiments were performed in accordance with the Guidelines for Animal Research from the National Institutes of Health and was approved by the Committee on Animal Research at İnönü University, Malatya, Turkey (2007/67).

### Histopathological examination

Samples prepared with a routine histological technique and sections were examined and scored using a Leica DFC280 light microscope and a Leica Q Win 1000 image analysis system (Leica Microsystems Imaging Solutions Ltd., Cambridge, UK). Assessment of tissue alterations in 20 different fields for each section was con-ducted by an experienced histologist who was unaware of the treatment. Gastric injury was scored by grading mucosal injury, congestion, haemorrhage and cell infiltration damage with a maximum score of 15. Scoring was performed for each parameter as follows: 0=no change, 1=mild, 2=moderate, 3=severe.

### Biochemical examination

The tissues were weighed for measurement of enzyme activities or levels, a PBS buffer with a ratio of 1/5w/v (pH: 7.4) was added and all the tissues were homogenized with packed cell volume, Kinematica, Status homogenizer (IKA-WERKE Ultra-Turrax, Germany) under ice isolation until they were all degraded. The resulting homogenates were sonified three times at 30-second intervals for 30 seconds with a VWR Scientific Branson sonificator (VWR Int. Ltd. Merch House Pool, UK). After the homogenization and sonification procedures, they were centrifuged at 12000 g for 15 min in an Ole Dich 157.MP micro centrifuge instrument (Ole Dich 157.MP micro centrifuge, Hvidovre, Denmark) at 4 °C. Thus, the supernatant in which enzyme and protein assays would be conducted was obtained. CAT, SOD and GSH-Px activities and GSH and malondialdehyde (MDA) levels were determined using the methods in the references (26-30).

### Statistical analysis

Statistical analyses were performed using the Kruskal-Wallis analysis of variance and Mann-Whitney U test on SPSS statistical software (SPSS for Windows version 13, SPSS Inc., Chicago, IL). All results were expressed as mean ± standard deviation (SD) (mean ± SD) and median (min-max). P<0.05 was considered statistically significant.

## RESULTS

### Macroscopic results

**Figure 1:**
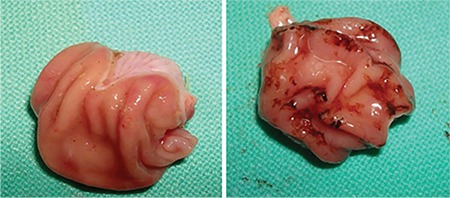
Macroscopic appearance of the inner gastric surface of a rat from the control group on the left and from the stress group on the right. Hyperaemia and haemorrhagic spots induced by stress are very obvious.

Hyperaemia and petechia were observed at the inner surface of the stomachs of rats exposed to stress (Figure 1).

### Microscopic results

**Figure 2:**
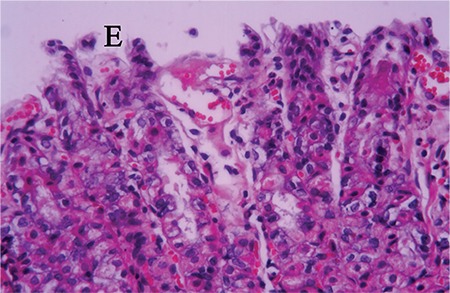
Stress group. Degeneration within the epithelium (E) and lamina propria was observed. HE; x20.

**Figure 3:**
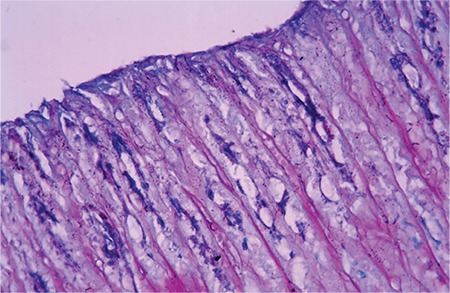
Stress + standard diet group. A weak staining is seen. PAS-AL; x40.

**Figure 4:**
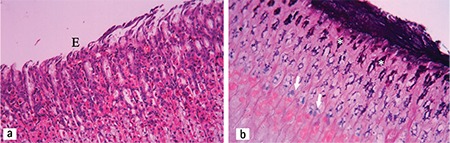
A. Stress + parsley-added diet group. Epithelial flattening (E) and degeneration are seen. HE; x20. B. Stress + parsley-added diet group. Positively stained thick mucus is observed on the surface. Violet colour in apical part of the glands (*) and blue colour (arrows) in the middle parts of the glands (arrows) staining was observed. PAS-Al; x20.

**Figure 5:**
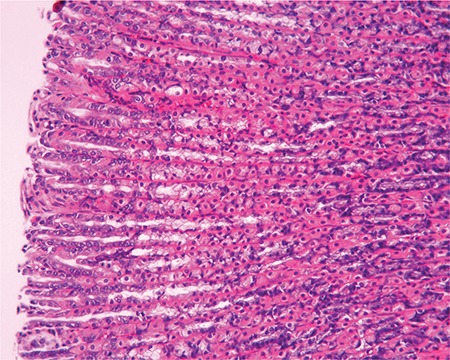
Stress + LPZ group. A normal histological appearance is seen. HE; x20.

Sections from the control group presented normal histology. However, several alterations indicating mucosal injury were detected in the sections of the rats exposed to stress. Degeneration and desquamation of the surface epithelium were observed (Figure 2). Congestion, haemorrhage and cell infiltration were detected in connective tissue. Periodic acid-Schiff (PAS)-Alcian blue staining on the surface and within the glandular lumen was pale and sometimes negative. In the sections obtained from the animals on a standard diet, mild mucosal damage was detected. PAS-Alcian blue staining on the surface and within the glandular lumen was also pale and sometimes negative (Figure 3). Sections from animals on the parsley-added diet were generally normal in histological appearance (Figure 4A), and the mucus layer on the surface was well preserved and very thick (Figure 4B). Sections of LPZ-treated rats very rarely showed epithelial desquamation (Figure 5). PAS-Alcian blue staining of the stress + LPZ group was positive in the cytoplasm of glandular cells, surface epithelial cells and on the surface. The mucus layer covering the epithelial surface was generally very thick.

The histopathologic damage score (HDS) was significantly higher in the stress group than the control group (p<0.05). The HDSs were 1.50±0.22 in control group, 7.00±0.57 in stress group, 4.00±0.17 in animals subjected to a normal diet, 3.83±0.30 in those subjected to a parsley-added diet and 2.33±0.33 in animals treated with LPZ. A statistically significant difference was found between the stress and stress + parsley-added diet groups (p<0.05), and the stress and stress + standard diet groups (p<0.05). Additionally, a statistically significant difference was found between the stress group and the stress + LPZ group (p<0.05). Among all groups except the control, the lowest HDS belonged to the LPZ-treated group. LPZ treatment was more effective than parsley-added diet administration (p<0.05).

### Biochemical results

The mean tissue MDA levels were also different between the groups. The MDA level of the stress group was significantly higher than that of the control group (p<0.05). The MDA levels were 0.73±0.05 in the control group, 0.88±0.02 in the stress group, 0.78±0.05 in the stress + standard diet group, 0.72±0.03 in the stress + parsley-added diet group and 0.71±0.04 in the stress + LPZ group. The MDA levels of the stress + parsley-added diet group and stress + LPZ group were lower than that of the stress group (p<0.05). The mean tissue MDA levels together with MDA are summarized in Table 1.

The GSH level and SOD and GSH-Px activities of the stress group were lower than those of the control group (p<0.05). In contrast, the CAT activity of the stress group was higher than that of the control group (p<0.05). The CAT activity of the stress + parsley-added diet group was higher than that of the control group (p<0.05). The lowest SOD activity was found in the stress group whereas the highest was found in the stress + parsley-added diet group. A significant difference was found between the stress group and the stress + parsley-added diet group (p<0.05). The GSH-Px values of the stress + standard diet group were higher than that of the stress + parsley-added diet group (p<0.05). The GSH levels of all groups were higher than that of the stress group. The GSH levels of the stress + parsley-added diet group were significantly higher than that of the stress group (p<0.05). The closest value to that of the control group was found in the stress + parsley-added diet group. The GSH levels and GSH-Px activities, and CAT and SOD activities of all groups, are shown in Table 1.

## DISCUSSION

Clinical and experimental studies have revealed that increased amounts of free radicals induce lipid peroxidation in various stress models (4,31,32,33). In this study, we also found a stress-induced increase in mean tissue MDA levels. The mean tissue MDA level and HDS of the stress group were significantly higher than in the control group (p<0.05).

In the present study, the tissue GSH level (p<0.05) together with GSH-Px (p<0.05) and SOD activities (p<0.05) were significantly decreased whereas CAT activity (p<0.05) was significantly increased in the stress group. Similarly to our results, decreases in tissue SOD activity (34-36), GSH-Px activity (37,38) and GSH level (30,39), and an increase in CAT activity (38), have been found. However, in some studies, no changes in SOD activity (38), increase in SOD activity (40) or decrease in CAT activity have been reported (39,40). Although the term ‘oxidative stress’ describes the insufficiency of the cellular antioxidant system against oxidant agents, the levels or the activities of the antioxidant enzymes have not been reported to be decreased in stress situations. The cells try to protect themselves initially via increased amounts of antioxidant enzyme production when they are exposed to any oxidant agent. This stage is probably a compensatory period. As a result of the damage of the organelles related to the production of the antioxidant enzymes, the amounts of the antioxidant enzymes decrease. In our study, SOD and GSH-Px activities and GSH levels were decreased but CAT activity was increased during the same stress period.

Conflicting results of studies emphasize the importance of detecting multiple antioxidant enzyme levels in order to evaluate the cellular antioxidant defence system. The response of each enzyme to oxidative agents may be different. Various factors, including the type and duration of the stress, the type of tissue and the species of the experimental animals, could be reasons for the conflicting results.

HSDs were 7.0±0.57 in the stress group (p<0.05) and 4.00±0.17 in the stress + standard diet group (p<0.05). We suggest that this recovery, even if no therapeutic agent had been applied, shows the self-repair capacity of the tissue that occurred within 7 days following the application of stress. Indeed, the tissue MDA level was decreased in this group in comparison to the stress group. Increases in SOD and GSH-Px activities and GSH levels are in fact indicators of time-dependent recovery of cellular antioxidant defence.

Parsley is a powerful antioxidant and free radical scavenger, especially due to its flavonoid content (14,15,20). Flavonoids show antioxidant, anti-inflammatory, anti-secretory, anti-proliferative and anti-ulcer effects in the gastrointestinal tract (41,42). To the best of our knowledge, the present study is the first to investigate the effect of fresh parsley on the gastric damage induced by stress. In fact, this study was inspired by, and based on, the experience of one of our authors whose gastric burning and pain induced by stress were relieved shortly after ingesting fresh parsley. In line with this experience, HDS and MDA levels were decreased in the stress + parsley-added diet group. The differences between the stress + standard diet and stress + parsley-added groups in particular emphasize the benefit of fresh parsley added to a daily diet. In different ulcer models, many plants like parsley containing tannins, sterols and flavonoids were found to protect gastric mucus and reduce MDA levels (17,43,44,45,46). Plants that contain flavonoids have been reported to increase CAT, GSH and SOD activities in stress-induced ulcers (37,38,39,40,41,42,43,44,45,46). In the present study, the mean CAT and SOD activities and GSH levels of animals from the stress + parsley-added diet group were higher than those of the stress and stress + standard diet groups. The increase in CAT activity in the stress + parsley-added diet group in relation to the stress + standard diet group was particularly noteworthy. The biochemical results show that parsley strongly supports the antioxidant enzyme system. Nielsen et al. (47) have reported increases in erythrocyte GSH reductase and SOD activities after nutrition with a parsley-rich diet for 2 weeks in a voluntary group consisting of 14 healthy people.

The mucus layer covering the epithelial surface of the gastrointestinal tract is an important protective layer (48). In our study, a weak staining was observed on the surface and glandular epithelium of the animals exposed to stress. In particular, the thickness of the superficial mucus is significantly reduced and absent in some areas. In contrast, in the stress + parsley-added diet group, PAS staining was very positive within the surface and glandular epithelium, and on the surface. This layer was found to be quite thick in some areas. Al-Howiriny et al. (17) found that mucus secretion was improved with the application of parsley. We suggest that the supportive effect of parsley on the gastric mucus barrier plays an important role in its beneficial effect on improving oxidative damage.

H_2_ receptor blockers and PPIs are commonly used drugs in the treatment of stress ulcers (10,12). LPZ is a PPI that inhibits the H+/ K+ ATPase enzyme in parietal cells. In experimental ulcer models, the therapeutic capacity of LPZ depends mainly on its suppressing effect on acid secretion and the amount of gastric pepsin (49,50) and on the effect of scavenging OH- radicals (51,52). Nakamura et al. (53) have demonstrated that LPZ increases microvascular and connective tissue regeneration in particular in the treatment of ulcers generated by ethanol.

In the present study, we also found a significantly reduced HDS in the stress + LPZ group compared to the stress group (p<0.05). Maiti et al. (54) and Blandizzi et al. (55) found LPZ effective in protecting gastric mucosa. The therapeutic effect of LPZ on gastric mucosal damage is found to be related to its mucus-increasing effect rather than its aciddecreasing effect (56,57,58). Nakamura et al. (53) found that the relationship between the structure consisting of mucus, cell debris and proteins, known as the “mucoid cap”, and myofibroblasts is important for gastric healing. Researchers concluded that acid suppression is important in the early stages of recovery with LPZ, but that the gastric mucosal defence mechanisms should be maintained for long-term recovery. In this study, we observed that LPZ administration increased the amount of mucus. This layer was found to be quite thick in some areas.

In the present study, LPZ reduced HDS and MDA levels, but increased SOD and GSH-Px activities and the GSH level compared to the stress group. CAT activity was higher than that of the control group. In ulcer studies, increased MDA and SOD values, and decreased CAT, GSH and GSH-Px values or decreased MDA and SOD values, and increased CAT, GSH and GSH-Px values have been reported (34,35,36,37,38,39,40,59,60). LPZ was shown to increase sulfhydryl compounds such as GSH in particular, and thereby prevent gastric damage (55,61). LPZ, indeed, is known to support the antioxidant system (52,59,60).

In conclusion, the addition of fresh parsley to the diet of experimental animals is effective in decreasing stress-induced gastric mucosal damage by supporting the cellular antioxidant enzyme system. It is exciting to realize that fresh parsley is more effective than LPZ in supporting tissue GSH levels and CAT activities. We suggest that a fresh parsley-rich diet together with classical drug treatment may accelerate the healing process.

## Figures and Tables

**Table 1:**
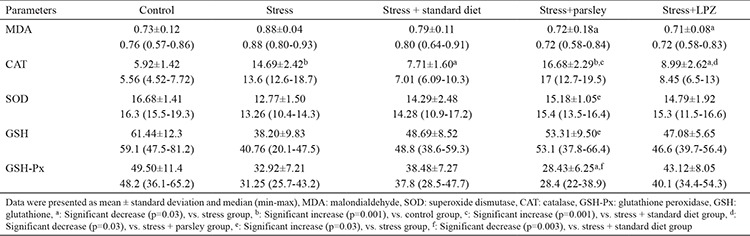
The levels of biochemical parameters of all groups
